# Decarbonization will lead to more equitable air quality in California

**DOI:** 10.1038/s41467-022-33295-9

**Published:** 2022-09-30

**Authors:** Shupeng Zhu, Michael Mac Kinnon, Andrea Carlos-Carlos, Steven J. Davis, Scott Samuelsen

**Affiliations:** 1 Advanced Power and Energy Program, University of California, Irvine, Irvine, CA 92697 USA; 2 Department of Civil and Environmental Engineering, University of California, Irvine, Irvine, CA 92697 USA; 3 Department of Earth System Science, University of California, Irvine, Irvine, CA 92697 USA

**Keywords:** Climate sciences, Environmental sciences, Energy science and technology, Risk factors

## Abstract

Air quality associated public health co-benefit may emerge from climate and energy policies aimed at reducing greenhouse gas (GHG) emissions. However, the distribution of these co-benefits has not been carefully studied, despite the opportunity to tailor mitigation efforts so they achieve maximum benefits within socially and economically disadvantaged communities (DACs). Here, we quantify such health co-benefits from different long-term, low-carbon scenarios in California and their distribution in the context of social vulnerability. The magnitude and distribution of health benefits, including within impacted communities, is found to varies among scenarios which reduce economy wide GHG emissions by 80% in 2050 depending on the technology- and fuel-switching decisions in individual end-use sectors. The building electrification focused decarbonization strategy achieves ~15% greater total health benefits than the truck electrification focused strategy which uses renewable fuels to meet building demands. Conversely, the enhanced electrification of the truck sector is shown to benefit DACs more effectively. Such tradeoffs highlight the importance of considering environmental justice implications in the development of climate mitigation planning.

## Abstract (web summary)

Air quality is found to be more equitable through two salient decarbonization pathways for California in 2050 with the relative justice of decarbonization scenarios quantified at the neighborhood level and the tradeoffs between pathways evaluated.

## Introduction

Current California environmental quality goals are deeply intertwined with the clean energy systems and fuels both deployed and evolving, to meet current and future energy demands. In particular, a dramatic reshaping of California energy systems and fuels is underway including transitions from fossil fuel combustion to low carbon technologies and fuels, with a priority on zero emission and renewable energy sources in nearly every emitting sector of the economy^1^. The transition cannot occur soon enough. California continues to experience challenges associated with degraded air quality including elevated concentrations of both ozone and fine particulate (PM_2.5_)^2^. Furthermore, California has established commitments requiring reductions in emissions of greenhouse gases (GHGs) including the original targets of 40 and 80% below 1990 levels by 2030^3^ and 2050^4^, and the more recent executive order “B-55-18^5^” requiring the state to reach carbon neutrality no later than 2045. California is already experiencing significant economic and health damages from the effects of climate change, including an increase in intensity and incidents of wildfires^6,7^ and deteriorating air quality^8,9^. Concerns over the damages brought by air quality and climate change are further exacerbated by the disproportionate shares both of these risks imposed on socially and economically disadvantaged populations^10^.

How these targets will be met is still uncertain due to the wide range of technologies, fuels, and other considerations that can provide GHG reductions. Electrification of fossil fuels depended sectors in tandem with electricity generated from renewable sources is certain to play a central role in California^11^. However, different strategies existed to achieve the same GHG reductions by adjusting the level of electrification penetration in different sectors. Further, resource availabilities can yield tradeoffs for allocating some low or net-zero carbon fuels (biofuels are a prominent example) across end-use sectors, particularly for those that are challenging to electrify^12^. One source of uncertainty is the future of the natural gas system, which represents an installed asset that could be managed in different ways to store and distribute low carbon gaseous fuels including renewable hydrogen and methane^13^. In this work, two different scenarios are considered to provide insight into the impacts of technology innovation, policy, and other drivers that will shape the evolution of energy sectors in California including a focus on the relative tradeoffs of decarbonizing the built environment and heavy-duty vehicles.

As GHGs are often co-emitted with various pollutant species including oxides of nitrogen (NO_x_) and PM_2.5_, meeting carbon targets will also provide improvements in regional air quality^14–16^, which have been shown to be sizeable in California^17–21^. Similarly, the public health benefits that result from air quality improvements are large and often comparable to the costs incurred from implementing mitigation strategies^22–24^, Zapata et al. for example report health savings of $11–20 billion annually from a comprehensive low carbon scenario in California^19^. However, health risks from exposure to degraded air quality are not uniformly distributed, and some regions and population segments bear a disproportionate share which often correlate with socially and economically disadvantaged communities (DACs)^25^. Further, many of the same communities suffer additional socio-economic risks that increase their exposure to air pollution damages, including employment and inadequate protection equipment^10^, as well as access to food and healthcare, and it is in these communities that health benefits from GHG mitigation efforts are most needed.

Although studies have shown low-income and underrepresented communities are more disadvantaged to climate change^26^ and air pollution^27^, only a few have evaluated the air quality implications of climate mitigation from an environmental justice perspective, and these are generally based on historical air quality in relationship to various climate policy frameworks^28–31^. Thus, there is a need for assessment of the distribution of air quality-driven health benefits that result from different long-term low carbon scenarios, which requires the quantification of health benefits via detailed and comprehensive air quality modeling. A limited number of studies utilizing similar approaches confirm moderately higher benefits within California disadvantaged communities relative to the general population as a whole^32–34^. However, these studies evaluated a single scenario which limits insights from comparing mitigation strategies in terms of technologies and fuels, e.g., the co-benefits of widespread electrification across the California economy^34^ or considered only one economic sector only, e.g., carbon neutrality in on-road vehicles^33^. The air quality co-benefits of different mitigation pathways were explored in Aas et al.^32^, however only health impacts from episodic modeling (two weeks in summer and winter) were quantified. Furthermore, and perhaps most importantly, all the studies discussed here provide a cursory assessment of environmental justice (EJ) implications generally represented by the summation and comparison of total benefits within disadvantaged communities relative to the California population as a whole^35^.Therefore, a need exists for the systematic evaluation of how reshaping energy systems to reduce GHG can best provide air quality health benefits within disadvantaged communities to support policy decisions regarding technology investment and other drivers of mitigation efforts.

In this work, the results from Aas et al.^32^ are taken as a starting point and utilizes annual air quality simulations and a novel framework for evaluating EJ air quality co-benefits to compare and contrast two different pathways for meeting California’s original 2050 GHG target (80% GHG reduction compares to 1990 levels). The two scenarios are selected to compare and contrast different outcomes for high building electrification and high truck electrification while using of the Nature Gas (NG) system to store and transmit renewable fuels as shown in Fig. S1. The resulting air quality improvements for each mitigation scenario relative to a Reference (REF) scenario are then quantified using the Community Multi-scale Air Quality Model (CMAQ)^36^. The public health benefits are then quantified and valued using the US Environmental Protection Agency’s Benefits Mapping and Analysis Program (BenMAP)^37^. Only the PM_2.5_ long-term health effects are considered here, as previous studies have shown the change in PM_2.5_ levels yields the majority of health benefits^38–40^, and it is also the most studied endpoint. Finally, an assessment of the distribution of health benefits from an EJ perspective is conducted based upon the rankings of community disadvantage in California from the CalEnviroScreen^25^ tool. The results provide insight for policy makers into how portfolios of mitigation strategies can be designed to attain maximum benefits in disadvantaged communities and can be used to guide investments that best improve public health in disadvantaged segments of the population.

## Results

### Technical summary

Here we developed a novel Lorenz-Curve based method to systematically evaluate and the efficacy of obtaining EJ improvements centered on the magnitude and distribution of air quality health co-benefits. This method is originally inspired by the Suits Index^41^, a variation of a Gini Index^42^, which is widely used as a measure of tax policy progressiveness. A larger Suits Index indicates a co-benefits distribution more favorite the disadvantaged communities and thus a higher EJ improvements efficacy. Usually, different climate policy could result to co-benefits with different magnitude at different regions. It is difficult to compare the efficacy of the policy in EJ improvement directly, as one policy might results to higher absolute co-benefits to DACs while still have an overall distribution favorite the less disadvantaged communities simply because it has a much larger total co-benefit. The advantage of this novel method is that it evaluates the normalized co-benefits distribution which sets an equal standard for policy evaluation regardless of the absolutes totals. This makes it possible to compare the policy EJ efficacy across different regions/sub-regions, between different policies, or even within one policy, as it is quite often to have distinct total co-benefits for different regions.

The two mitigation scenarios used in this work, one with substantial electrification of residential and commercial building appliances (referred to hereinafter as “building electrification”) and another where much higher electrification penetration of medium and heavy-duty trucks is assumed (“truck electrification”). For the truck electrification scenario, gaseous fuels continue to provide most building energy, but are derived from renewable rather than fossil sources. Furthermore, most non-electrified trucks in both scenarios are transition to compressed natural gas (CNG) as fuel source. This tradeoff is designed to assess different pathways for decarbonizing the existing natural gas system in California as building appliances consume the bulk of current natural gas demand. Therefore, while the two scenarios do provide interesting differences to compare and contrast, they are not designed from an air quality perspective, and this should be considered in interpreting the results.

### Air quality and health co-benefits

**Fig. 1:**
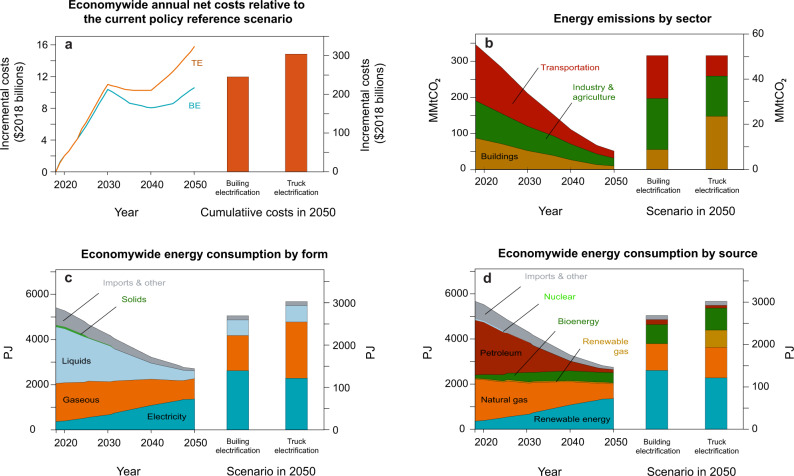
Projected cost, GHG and energy consumption for scenarios. (Adapted from Aas et al.). **a** Economy-wide incremental annual and total costs for each scenario relative to the REF (BE for building electrification and TE for truck electrification). **b** CO_2_ emissions from energy production by industry sector. Both scenarios achieve the same level of emissions by 2050. **c** Economy-wide energy consumption by form. Electricity includes solar, wind, hydroelectric, and geothermal generation. Gaseous includes natural gas, SNG, CHP, biomethane, and hydrogen. Liquids include liquid petroleum and liquid biofuel. Solids include nuclear and biomass. **d** Economy-wide energy consumption by source. Renewable energy includes solar, wind, hydroelectric, and geothermal energy. Truck electrification includes SNG and hydrogen. Natural gas also includes CHP. Biofuel includes biomass, liquid biofuel, and biomethane. The left portion of **b**–**d** corresponding to the building electrification.

Figure 1 presents the economy wide costs, GHG emissions, and energy distribution for the two mitigation scenarios as reported in Aas et al.^32^. Figure 1a shows a lower incremental cost for the building electrification scenario ($10.6 billion 2018$) relative to the truck electrification scenario ($15.8 billion 2018$). By 2050, GHG emissions will be reduced by 88% for transportation, 78% for industry and agriculture, and 90% for buildings in the building electrification scenario, and by 92% for transportation, 79% for industry and agriculture, and 67% for buildings in the truck electrification scenario (see Fig. 1b). More energy is delivered in the form of electricity in both scenarios, but it increases from 7% in 2018 to 40% in the truck electrification scenario and to 52% in the building electrification scenario, whereas gaseous fuels reach 44 and 31% of energy use in these scenarios, respectively (Fig. 1c). Meanwhile, energy from petroleum decreases from 43% to <5% 2018–2050 in both scenarios, and renewable sources increase dramatically from 6% to >23%, with bioenergy increasing from 2.5 to 17%.

**Fig. 2:**
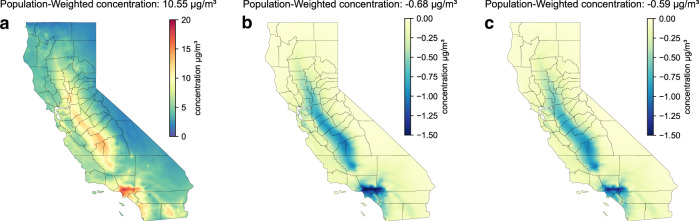
Baseline air quality and attained improvements from carbon mitigation efforts. **a** Baseline annually averaged PM_2.5_ concentrations for the reference scenario and annually averaged PM_2.5_ concentration reductions from **b** the building electrification scenario and **c** the truck electrification scenario.

**Fig. 3:**
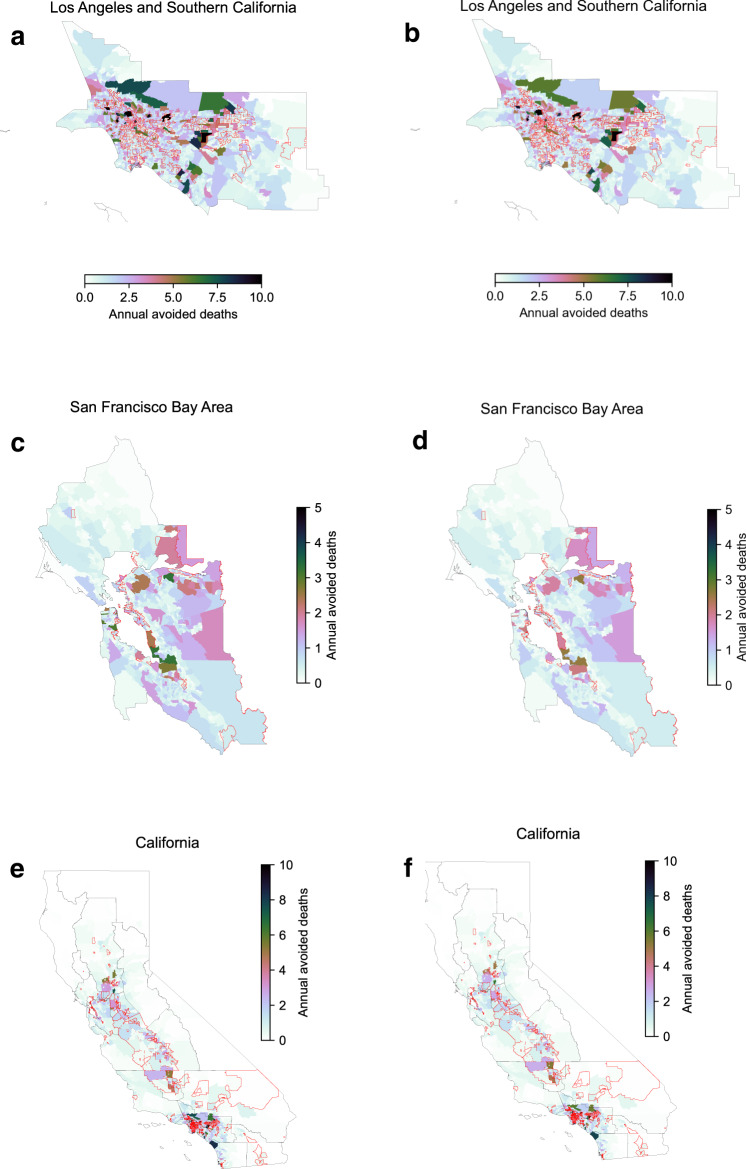
Spatial distribution of public health benefits. Annual avoided deaths at the census tract level for **a**, **c**, **e** building electrification scenario and **b**, **d**, **f** truck electrification scenario relative to reference scenario for the two most populated air basin and the whole state; the red line highlights the location of disadvantaged communities.

Figure 2a shows modeled results of annually averaged PM_2.5_ concentrations in 2050 for the REF baseline with a population-weighted concentration of 10.55 μg/m^3^. With respect to the current annual PM_2.5_ standard of 15 μg/m^3^, high baseline PM_2.5_ concentrations occur within the San Joaquin Valley (SJV) and the South Coast Air Basin (SoCAB). Figure 1b, c show PM_2.5_ reductions occurring in the building electrification and truck electrification scenarios, respectively. In general, similar spatial distributions of PM_2.5_ improvements occur for both scenarios, with the reductions most evident within the SJV and SoCAB. Population-weighted PM_2.5_ benefits are somewhat greater under the building electrification scenario (−0.68 μg/m^3^) than the truck electrification scenario (−0.59 μg/m^3^). Although the difference looks small numerically, while, due to the huge population of California (>44 million in 2050), the accumulated exposure difference between two scenarios is still significant, which is reflected in the following health impact assessment. Compares to the 5 μg/m^3^ exposure guidelines recommended by the world health organization^43^, significant reductions existed over some of the most disadvantaged communities (See Fig. 3) in the SoCAB region (1.72 μg/m^3^ and 1.46 μg/m^3^ for building and truck electrification scenarios respectively). The CMAQ model used in this study can identify such level of differences accurately as demonstrated in previous studies^8,44–46^.

**Fig. 4:**
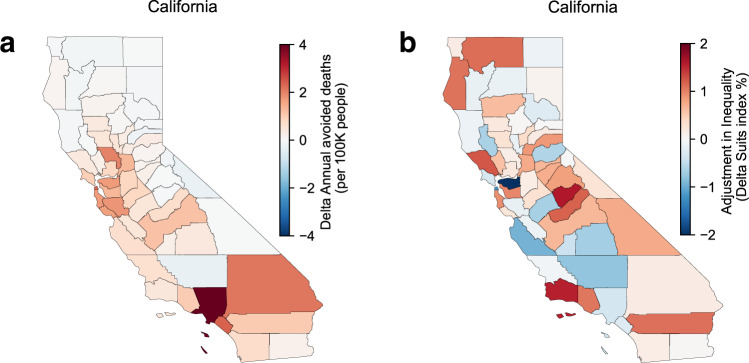
Spatial distribution of public health benefits. The spatial distribution of delta (building electrification-truck electrification) avoided deaths (**a**) and Suits Index (**b**) by county (a negative delta Suits Index indicates higher health benefits allocation towards disadvantaged communities in the truck electrification scenario compares to the building electrification).

Figure 3 compares census tract-level health benefits under the different energy-emissions scenarios. As with air quality improvements, the spatial distribution of health benefits (Fig. 3e, f) is broadly similar between the decarbonization scenarios (Fig. 2a, b) and concentrated in the SoCAB and SJV. Statewide, total avoided deaths are ~6100 under the building electrification scenario and ~5300 under the truck electrification scenario. Focusing on disadvantaged communities (Fig. 3), which include most of the SJV and many areas of SoCAB (e.g., parts of Los Angeles, Riverside, and San Bernarndino counties), the building electrification scenario would avoid ~1800 deaths in comparison to ~1500 avoided by the truck electrification scenario, or 28.9% and 29.3% of statewide totals in each case, respectively. Figure 4a shows comparison of population averaged avoided mortality rate at the county level between two scenarios, with the building electrification scenario providing higher health benefits than the truck electrification scenario in most counties, particularly in densely populated urban areas. Conversely, the truck electrification scenario provides greater benefits to some rural areas, especially in Northern California.

### Impacts on environmental justice

**Fig. 5:**
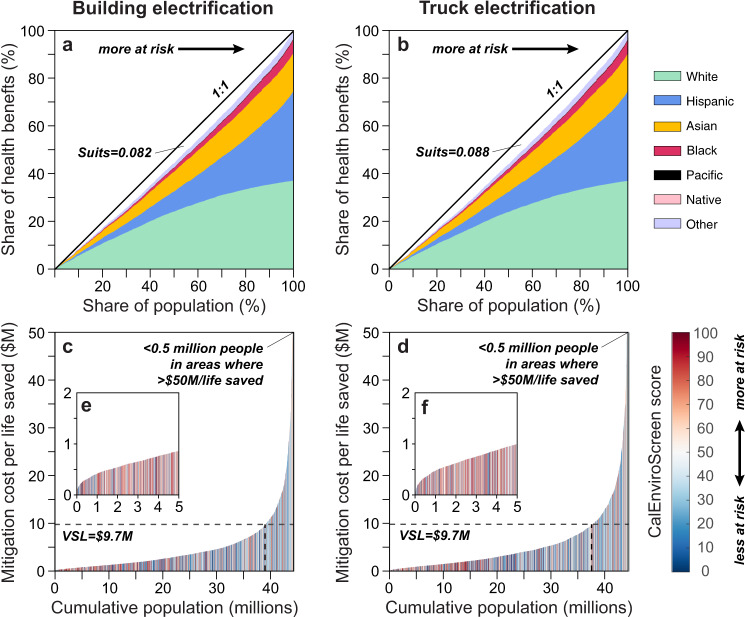
Distribution of health benefits and mitigation costs per life saved. Health benefits distribution across census tracts in 2050 for the **a** building electrification scenario and **b** truck electrification scenario. Census tracts are sorted by increasing CalEnviroScreen 4.0 score and colored by7 racial/ethnicity accumulated attributions. The 1:1 line of equal distribution is plotted for comparison. The Suits Index for each scenario is shown. Scenario distributions are nearly identical, with in general increasing non-white population for more disadvantaged communities. The building electrification scenario has a smaller Suits Index than the truck electrification scenario, indicating less progressive benefits distribution towards disadvantaged communities. Mitigation costs per life saved for each census tract in 2050 (in 2018 dollars) for the **c** building electrification scenario and **d** truck electrification scenario. Census tracts are sorted by increasing mitigation costs per life saved and colored by the CalEnviroScreen 4.0 index. To improve the color clarity, the first 5 million population on the left side of the plots are zoom out in corresponding subpanel (**e**) building electrification and **f** truck electrification. The estimated VSL of $9.7 million is plotted for comparison. In all scenarios, most tracts have values below the VSL, with the building electrification scenario having the most tracts below.

Figure 5a–b shows the statewide Lorenz Curves for each scenario, with California census tracts sorted from left to right by low to high environmental risk^25^ and colored by race and ethnicity accumulated attributions. The relationship between environmental risk and non-white population attribution is evident with the slowdown of white attribution and rapid growth of non-white attributions towards the right end (more disadvantaged tracts), especially for Black and Hispanic groups. However, the distribution of health benefits is not even across the population and are somewhat different in the two scenarios. In both, the most disadvantaged tracts experience a disproportionate share of the health benefits, but this disproportionality is somewhat more pronounced in the truck electrification scenario, as indicated by the greater Suits Index (i.e., the area between the 1:1 line and the bars; Fig. 5b). In other words, disadvantaged communities benefit most from air quality improvements of decarbonization, but the ratio is more favorable to those communities in the truck electrification scenario relative to non-disadvantaged communities. However, this should again be considered within the result of the larger total health benefits the building electrification scenario achieves within impacted communities. Additional data dispersity analysis with the 0–1 normalized census track level health benefits also shows a less dispersed (more uniform) probability density distribution for the truck electrification scenario (with a standard division *σ* = 0.0384) compared to the building electrification (*σ* = 0.0386), which is consistent with our Suits Index results.

If the Suits Index is instead calculated at the county level, the truck electrification scenario is more favorable to disadvantaged communities in 21 (36%) of California counties, and especially in populous areas such as Los Angeles and San Francisco County, suggesting the benefits from heavy-duty electrification are particularly notable in those regions (Fig. 4b). However, the building electrification still have higher total co-benefits in most of those counties, expect for Kern County where both the total co-benefits and EJ efficacy is higher in truck electrification. Although positive Suits Index is calculated at the state level for both scenarios, indicating an overall EJ improvement statewide. However, the Suits Index at the county level shows negative values for 29 and 28 counties in the building and truck electrification scenarios respectfully (Fig. S4), with Merced the only county switch side. A negative index indicates the overall co-benefits distribution within those counties is not favoriting disadvantaged communities. Supplementary Figs. S5 and S6 further compare health benefits allocation between the scenarios for the state and Los Angeles County. Inside Los Angeles County, truck electrification has higher benefits allocation around highways or traffic hubs like downtown Los Angeles, Glendale, and San Pedro Bay Ports, or downwind mountain foothills like Pasadena, Bradbury, and Citrus, where pollutants are easily trapped. Conversely, benefits are higher in the building electrification scenario for regions with large numbers of buildings or downwind of large urban populations including west Los Angeles, and most of the coastal regions. Outside of Los Angeles County, truck electrification has higher benefits allocation in most of SJV where the highway 5 is located, and some of the highest difference appears in Kern County and San Diego County (Fig. S6). While the building electrification generally have higher allocation around the San Francisco Bay area and part of the SoCAB. These results demonstrate the complexity of understanding air quality impacts within an environmental justice framework. Furthermore, it should be noted that both scenarios achieve important equity benefits relative to the reference scenario and the differences discussed here between them are relatively minor.

Total mitigation costs in 2018 dollars for each scenario were calculated from economy-wide annual net costs relative to the reference scenario by Aas et al.^32^ Dividing the total mitigation cost of each scenario by the projected population in 2050 (i.e., per capita costs assuming each California resident pays an equal share of the costs), we estimate the implicit cost per life saved in each census tract. Figure 4c compares cumulative mitigation costs against cumulative population in 2050, with a valuation of statistical life (VSL) of $9.70 million (2018$) also plotted for comparison (Roman and Robinson, 2016). In most census tracts, the mitigation cost per life saved is less—and sometimes much less—than the VSL (See also Table 1). Statewide, applying the VSL of $9.70 million per life, the economic value of deaths avoided in the building electrification and truck electrification scenarios in 2050 is $59 billion and $51 billion, respectively, which less costs translate to net total social-economic benefits of $49 billion and $35 billion, respectively.

**Table 1:** Cost/benefit analyses

	Building electrification scenario	Truck electrification scenario
Population with net benefit	90%	87%
Census tracts with net benefit	88%	84%
Disadvantaged communities with net benefit	95%	94%

Percentage of population, census tracts, and disadvantaged communities with mitigation costs per life saved values less than the VSL.

## Discussion

Despite the substantial costs estimated to meet long-term carbon goals in California, the results show that the potential air quality co-benefits alone are sufficient to provides net benefits for both scenarios considered. However, it should be considered that the bulk of the health benefits valuation is associated with a willingness-to-pay through the VSL, rather than a direct cost savings. For air quality co-benefits, the building electrification scenario (−0.68 μg/m^3^) outperforms the truck electrification scenario pathway (−0.59 μg/m^3^) with higher population-weighted PM_2.5_ exposure reductions. The same performance also holds for the total social-economic benefit: net benefits of the building electrification scenario are $14 billion more than the truck electrification scenario. Also, 90% of the population benefits in the building electrification scenario, whereas only 79% of the population benefits under the truck electrification scenario. However, when the distribution of benefits among groups with different socio-economic vulnerability, the truck electrification scenario favors disadvantaged communities marginally more than the building electrification scenario (Fig. 4) due to the enhanced heavy-duty vehicle electrification. Such trade-offs point to the importance and challenges of including environmental justice when evaluating plans for climate mitigation.

Our analysis is subject to several important caveats and limitations. First, we consider only a few decarbonization scenarios and stop short of the net-zero emissions goal that has been targeted by recent policies. In particular, the two scenarios assessed were designed to compare and contrast tradeoffs between scenarios meeting different outcomes for the utility gas system and therefore do not represent the air quality benefits of all possible measures. Meteorological conditions are also kept constant in this study; because climate change is likely amplify the air quality impacts of emission mitigations^8^, we may thus be underestimating the air quality benefits of the scenarios. Finally, our epidemiological analysis only takes into account the long-term effects of PM_2.5_ exposure (usually >90% of the health valuation in California^6,35,47^), thus neglecting additional effects of other pollutants such as ground-level ozone and air toxics, this also doesn’t take into account acute effects of different pollutants.

Nonetheless, our results suggest that meeting California climate goals will generally improve air quality and public health. Moreover, given the uneven distribution of air pollution in the current system, there will be disproportionate benefits for socially and economically disadvantaged communities regardless of the technological pathway of decarbonization. However, policies that affect the composition of technologies and fuels in major energy sectors may be designed to prioritize these equity improvements, for example by identifying strategies that achieve regional or sub-regional air quality improvements at the community level. In this work, both the electrification of buildings and transitions to zero emission heavy-duty vehicles are shown to offer notable benefits to impacted communities, however the benefits accrue differently. For example, aggressive building electrification can attain large total benefits, but equivalent levels of electrification targeting a reduction in emissions from heavy-duty vehicles could yield higher benefits among disadvantaged communities. Yet the overall level of health benefits of a policy may be at odds with more equitable distributions of those benefits: only by quantifying both the magnitude and distribution of benefits can decision makers balance these goals. It should also be considered that the challenge of meeting the current California goal of carbon neutrality by 2045 will require large emissions reductions by all sectors of the economy. Within that context, both the electrification of buildings and heavy-duty vehicles should be prioritized due to the benefits shown here.

## Methods

### Scenario design

In this study, two climate mitigation scenarios meeting an 80 percent GHG reduction by 2050 from 1990 levels and one “Reference” scenario (REF) developed using the E3 PATHWAYS model were taken from Aas et al.^32^. This work was initiated prior to the 2018 executive order of carbon neutrality and therefore the scenarios do not meet that target. However, the mitigation measures considered here will be needed, potentially in addition to other approaches such as negative emissions technologies, and the results still provide important insights.

The two scenarios are designed primarily to compare and contrast the reliance on key decarbonization strategies, including residential and commercial building electrification, biofuels, and low carbon fuels for heavy-duty vehicles (HDV). A key aspect of the work is to evaluate tradeoffs between the use of electrification and truck electrification to decarbonize residential and commercial buildings.

First, the REF Scenario assumes only policy commitments included in the 2017 Scoping Plan Update^1^ and the “zero-carbon retail sales” interpretation of SB 100^48^, and does not meet the 2030 and 2050 California GHG goals. Previous work has identified electrification as a lower-cost and lower-risk strategy to decarbonize the California buildings sector, due largely to cost and resource supply limitations associated with the various truck electrification pathways including biomass and biogas pathways and electrolytic fuel production^11^. Therefore, in this work the “building electrification” scenario assumes nearly complete decarbonization of residential and commercial buildings by 2050 through the widespread electrification of space heating, water heating, cooking, etc. The building electrification scenario further assumes the moderate electrification of HDV through battery electric and hydrogen fuel cell vehicles, and transitions to compressed natural gas (CNG) of most non-electrified diesel trucks as an air quality mitigation measure. Limited biofuels are produced and are largely allocated with remaining fossil fuels in transportation and industry.

In contrast, the “truck electrification” scenario assumes a continued reliance on gas appliances supplied by pipeline gas for building energy but assumes the blending of renewable hydrogen and RNG into the natural gas supply and the deployment of high efficiency furnaces and water heaters. However, additional GHG reductions are still needed to reach 2050 targets and those are achieved primarily by the greater deployment of battery electric and fuel cell HDV relative to the HBE scenario. Biofuel and fossil fuels are largely used in buildings, e.g., 56% of the pipeline natural gas delivered remains fossil.

Both the building electrification and truck electrification scenarios assume a range of additional decarbonization measures that reduce pollutant emissions relative to the REF scenario including a reliance on renewable electricity, high electrification of light-duty vehicles buses, and off-road equipment, reductions in demand for petroleum refining, etc. However, those are held constant between the building and truck electrification scenarios and the criteria pollutant and air quality differences driving the results in this work result almost entirely from the assumptions regarding diverging decarbonization of buildings and HDV.

Table 2 summarizes key metrics for those three scenarios and more details regarding the scenario design can be found in the supporting information (Table S1) to this work and Aas et al.^32^.

**Table 2:** Scenarios summary of key metrics

Sector	Reference (REF)	Building electrification	Truck electrification
GHG emissions reduction	Does not meet state climate goals	40% by 2030 80% by 2050	40% by 2030 80% by 2050
Building electrification	None	High: 91% of energy consumption by 2050	Low: 49% of energy consumption by 2050
Light-duty vehicle electrification	Medium	High: 100% Sales by 2035	High: 100% Sales by 2035
Medium-duty battery electric trucks	None	Medium: 39% Sales by 2040	High: 71% Sales by 2040 and 91% by 2050
Zero-emission heavy-duty trucks: Battery and Hydrogen Fuel Cell	None	Medium: 31% sales by 2040 and 34% by 2050	High: 67% Sales by 2040 and 69% by 2050
Advanced low-NO_x_ CNG trucks	Displace some diesel trucks	Displace most non-electrified diesel trucks	Displace most non-electrified diesel trucks

Adapted from Aas et al.^32^ 2050.

### Air quality and public health benefits assessment

A comprehensive modeling method was used to quantify the air quality and public health co-benefits that accrue from the mitigation measures contained within each scenario relative to the REF scenario. First, the anthropogenic emissions are resolved based on the 2012 estimates from the California Air Resources Board (CARB) and control factors associate with each 2050 scenario using the Sparse Matrix Operator Kernel Emissions (SMOKE)^49^ modeling system. Figure S2 shows the reduction in air pollutant emissions for each scenario. Next, a chemical transport model: Community Multiscale Air Quality Modelling System (CMAQ, v5.2)^50^ is used to simulate atmospheric pollutant concentrations annually in 2050 for each scenario. The simulation domain covers entire California with a 4 km by 4 km horizontal resolution (Fig. S1). CMAQ is widely used for air quality assessment purposes including regulatory compliance and atmospheric research^51,52^ The SAPRC-07 chemical mechanism^53^ is used for gas-phase chemistry, and the AERO6 module^54^ is used to estimate aerosol dynamics. The Advanced Research Weather Research and Forecasting Model (WRF-ARW, 3.7) is used to produce meteorological conditions from the (Final) Operational Global Analysis data^55^. The boundary conditions come from Model for Ozone and Related Chemical Tracers (Mozart v4.0)^56^. While simulations are conducted using anthropogenic emissions representing 2050, the boundary and meteorology conditions are held constant and the impacts of future concentration drivers including transported pollution and climate are not considered. Finally, the environmental Benefits Mapping and Analysis Program—Community Edition (BenMAP-CE)^37^ is used to quantify public health benefits from improvements in PM_2.5_ for the building electrification and truck electrification scenarios relative to the REF scenario closely following methods used in analogous studies for California^57^. Population projections are obtained from demographic data^58^ at the census tract level. Baseline incidence rates for mortality and morbidity are estimated from public administrative records where feasible and projected from US Census Bureau data^59^. Concentration-response functions are selected based on suggested criteria from a systematic review of the epidemiological literature^60^ and a value of a statistical life (VSL) of $9.7 million in 2018 dollars is used^61^. Impacts are reported here for avoided incidence of premature mortality associated with long-term exposure to PM_2.5_. The concentration-response functions used for the health impact analysis is based on the study of Krewski et al^62^ and Jerrett et al^63^, and results are merged using the “Random and Fixed Effects” pooling method provided in BenMAP.

### Mitigation cost analysis

The mitigation costs for each scenario are taken from Aas et al.^32^ and were estimated by E3 using the California PATHWAYS model. PATHWAYS is an energy and infrastructure model that can be used to assess the cost and GHG emissions of California’s energy demand and supply decisions designed to achieve long-term climate targets^64^. PATHWAYS is used extensively in California to develop and assess economy-wide scenarios meeting climate policy targets using differing combinations of mitigation measures^11,65–67^. Given the focus on decarbonizing natural gas, a detailed technoeconomic assessment for each truck electrification pathway considered was conducted including costs (energy, capital, and feedstock) and resource potential as described in Appendix E of the referenced report^67^ and used to update PATHWAYS for this work. The economy wide costs estimated by PATHWAYS represent a total resource cost that includes all direct energy system costs within the California economy resulting from fuel consumption and from capital costs from energy infrastructure associated with purchase of building appliances or vehicles, as well as incremental energy efficiency or fuel-switching capital costs. To calculate mitigation cost for each census tract, it is assumed that costs to each individual are equally distributed across the California population. The mitigation cost for each census tract is then estimated by calculating the sum of individual costs within each census tract.

### Environmental justice assessment

Economist Daniel B. Suits^41^ initially proposed the Suits Index as a measure of tax progressiveness. Here, the Suits Index is used as an indicator of progressivity in public health benefits distribution among communities with different environmental justice statuses. Figure S3 illustrates how the Suits Index is calculated to assess each mitigation scenario with the *x*-axis corresponding to the socio-economic status of a census tract ranked by CalEnviroScreen 4.0^25^ where higher rankings are considered more disadvantaged disadvantaged to environmental hazards. The *y*-axis represents the cumulative percentage of total environmental benefits that accrue from air quality improvements resulting from the deployment of mitigation strategies within each scenario. OB represents a proportional or equal distribution of environmental benefits among all communities. At the same time, OCB shows a distribution in favor of disadvantaged communities, and ODB shows a distribution favoring the general population of non-disadvantaged communities. The Suits Index is the ratio between the area that a Lorenz-Curve (OCB or ODB) deviates from the proportionality line (OB) and the area given proportionality (between OB and OAB). For a curve (OCB) under the proportionality line, the area is considered positive, so its Suits Index will be positive. For a curve (ODB) above the proportional line, the area is deemed negative, so its Suits Index will also be negative. The Suits Index is a value between −1 and 1, with 0 indicating for equal distribution, positive values for progressive distribution, and negative for regressive distribution.

In this study, the CalEnviroScreen 4.0 environmental justice screening tool developed by California’s Office of Environmental Health Hazard Assessment (OEHHA) is employed to rank all California communities at the census tract level (from 0 to 100). CalEnviroScreen identifies communities risked by a disparate share of air pollution in addition to socioeconomic and health challenges that increase their risk to environmental health effects. CalEnviroScreen ranks each of the state’s 8000 census tracts according to multiple endpoints associated with pollution, environmental quality, and socioeconomic and public health conditions. Organizations ranking within the final 25% (score ≥ 75) are considered disadvantaged communities. Health benefits estimated from the air quality co-benefits of different technology pathways are used for the environmental benefits. The MATLAB v9.8 R2020a is used for data processing, visualization, and suit index calculations.

### Reporting summary

Further information on research design is available in the Nature Research Reporting Summary linked to this article.

## Supplementary information

Supplementary Information

Reporting Summary

## Data Availability

All data that support the findings of this study are present in the paper and the supplementary materials, and additional data are available in a publicly accessible repository. The Supplementary Information contains schematic diagram of mitigation pathways, table of scenarios summary of key metrics, emission reductions, conceptual diagram of suits index, and detailed EJ and health benefits allocation comparison for each scenario. The air quality data at 4 km x 4 km resolution and the health benefits data at the census tract level can be accessed through the public link: 10.17605/OSF.IO/S5RQK.

## Code availability

All codes regarding the Lorenz curves and suits index calculation can be accessed through the public link: 10.17605/OSF.IO/S5RQK.
