# Supplementary material for: Decarbonization will lead to more equitable air quality in California
Source: Nat Commun. 2022 Sep 30;13:5738. doi: 10.1038/s41467-022-33295-9 (PMC9525584; doi:10.1038/s41467-022-33295-9)
Supplement: Supplementary file 1 — Supplementary Information [file 41467_2022_33295_MOESM1_ESM.pdf]

Supplementary Information

Zhu et al.

Decarbonization will lead to more equitable air quality in California

Shupeng Zhu[1], Michael Mac Kinnon[1], Andrea Carlos-Carlos[3], Steven J. Davis[2, 3],

and Scott Samuelsen[1*]

[1] *Advanced Power and Energy Program, University of California, Irvine, CA 92697, USA*
[2] *Department of Earth System Science, University of California, Irvine, Irvine, CA 92697*
[3] *Department of Civil and Environmental Engineering, University of California, Irvine, Irvine, CA 92697*

[*]corresponding author: gss@apep.uci.edu

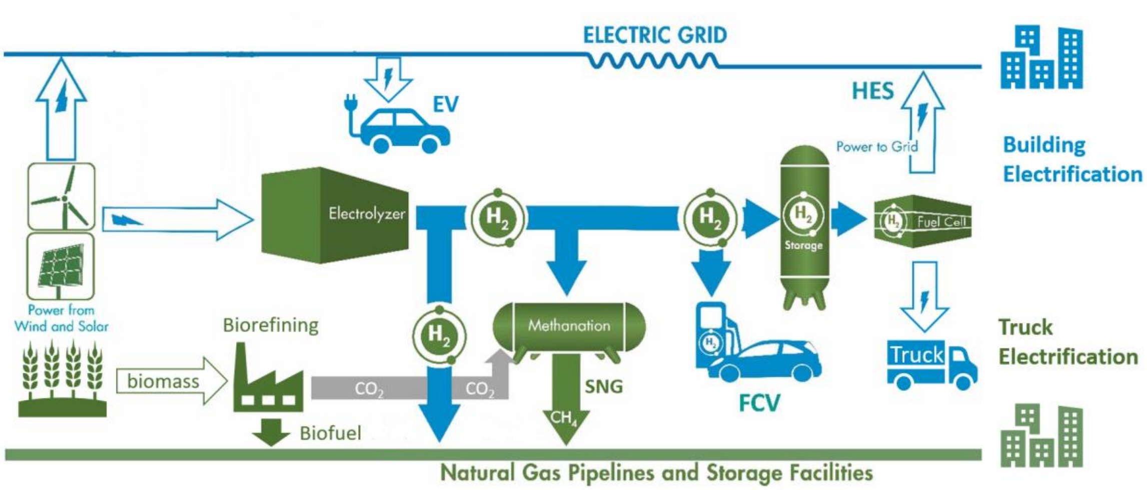

Figure S1 | Schematic diagram of renewable technologies adopted in two different climate mitigation pathways.

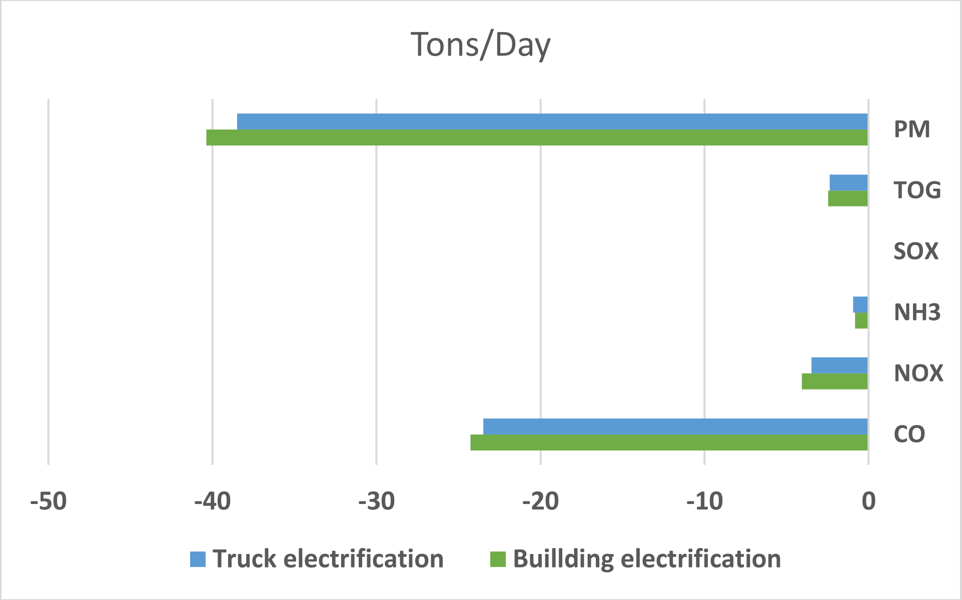

Figure S2 | Pollutant emission reductions relative to the REF scenario for the truck and building electrification scenarios.

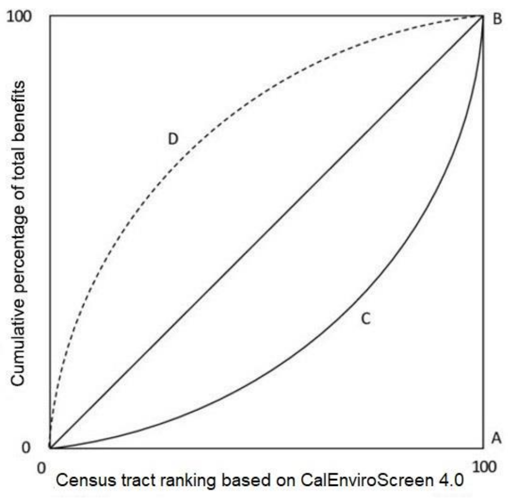

Figure S3 | Overview of the Suits Index used to assess health benefits distribution within DAC.

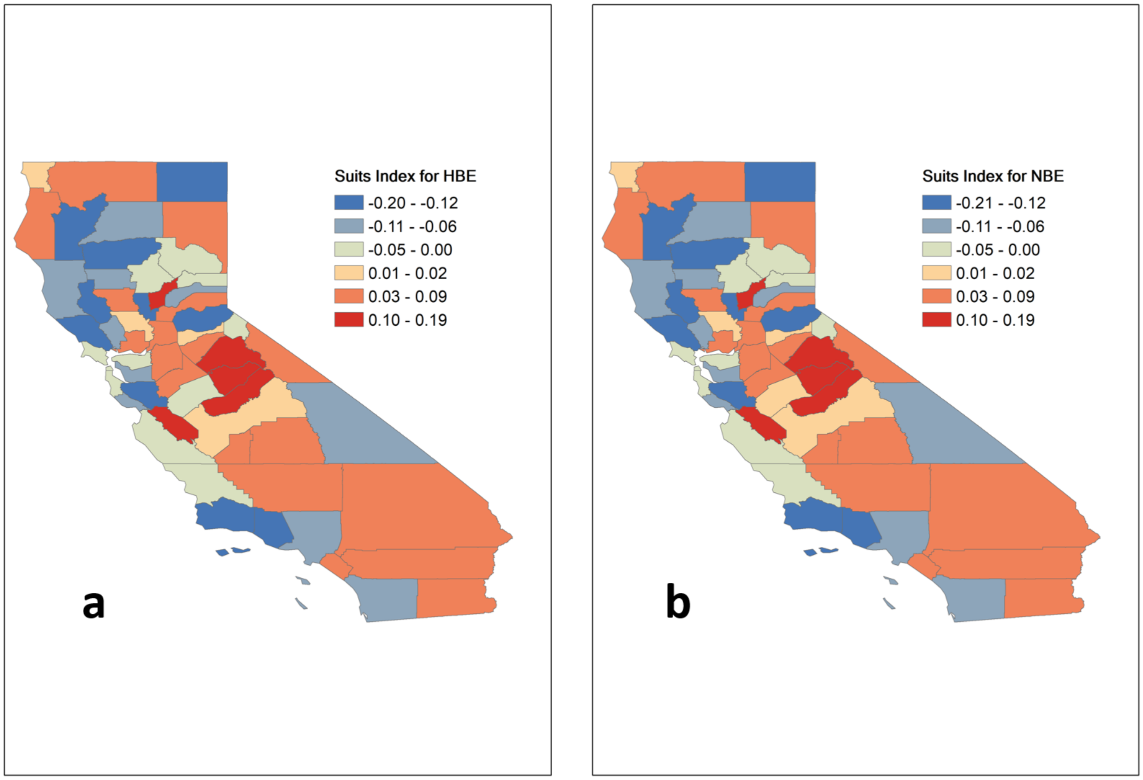

Figure S4 | County level Suits Index. Spatial distribution of Suits Index by county for (a) building electrification and (b) truck electrification.

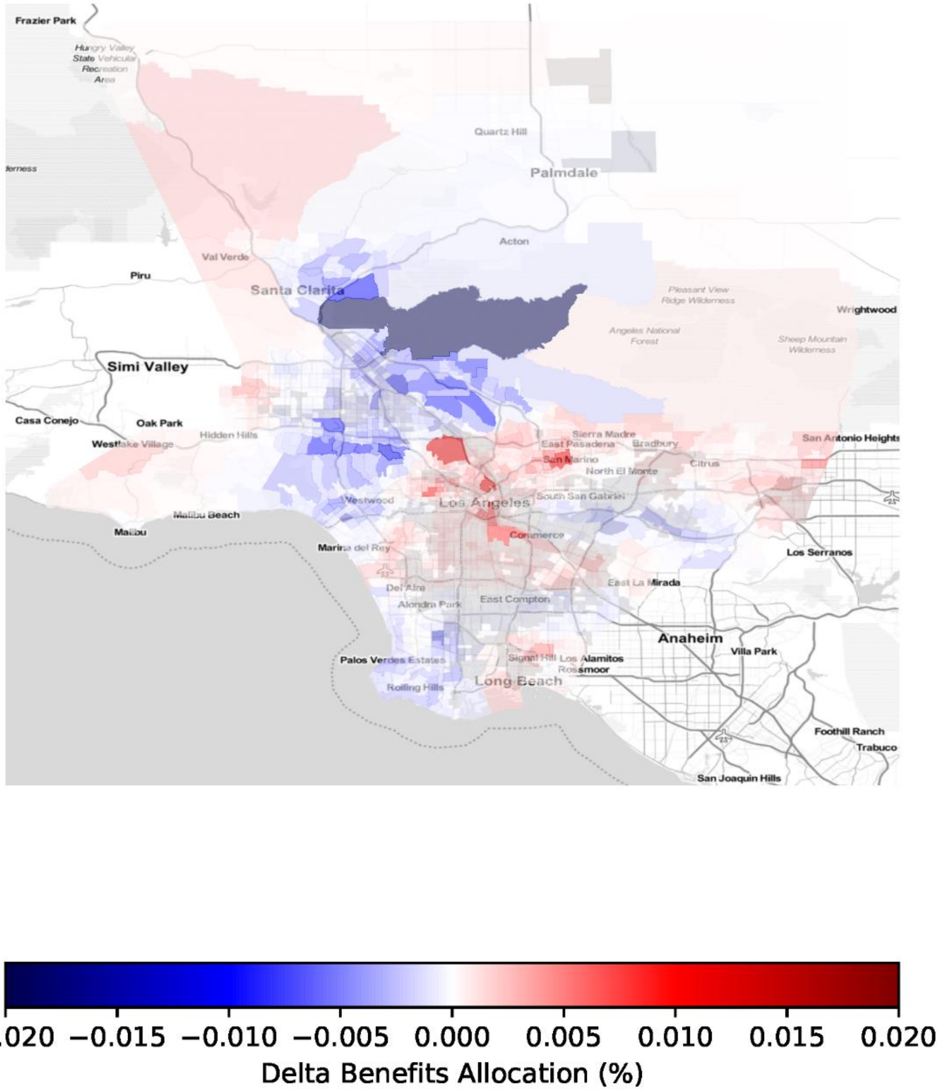

Figure S5 | Comparison of Health Benefits within Los Angeles County. Spatial distribution of the allocation difference between truck and building electrification. Positive value means higher allocation percentages of health benefits in the truck than building electrification. Grey region marks the DACs.

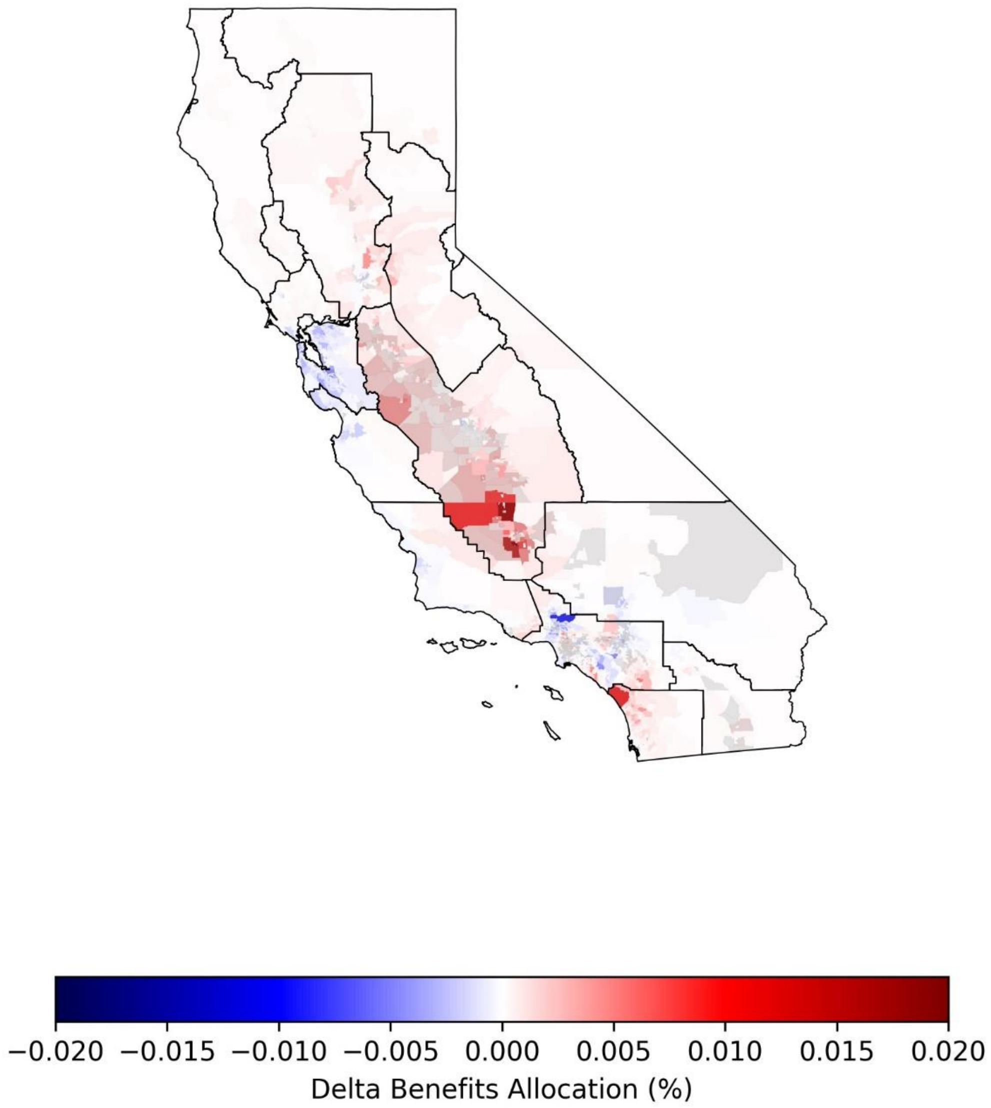

Figure S6 | Delta benefits allocation for California. Spatial distribution of the allocation difference between truck and building electrification. Positive value means higher allocation percentages of health benefits in the truck than building electrification. Grey region marks the DACs.

Table S1 | PATHWAYS Scenarios Summary of Key Metrics. Adapted from Aas et al.

**Sector**	**Reference (REF)**	**Building electrification**	**Truck electrification**
GHG Emissions Reduction	Does not meet state goals	40% by 2030<br>80% by 2050	40% by 2030<br>80% by 2050
Building Electrification	None	High: 91% of energy consumption by 2050	Low: 49% of energy consumption by 2050
Electricity	BAU	95% zero-carbon electricity generation by 2050	95% zero-carbon electricity generation by 2050
Pipeline Biomethane	None	25%	16%
Pipeline H_2	None	None	7%
Pipeline SNG	None	None	21%
Advanced Biofuels	71 TBTU	478 TBTU	533 TBTU
Light-Duty Vehicle Electrification	Medium	High: 100% Sales by 2035	High: 100% Sales by 2035
Medium-duty battery electric trucks	None	Medium: 39% Sales by 2040	High: 71% Sales by 2040 and 91% by 2050
Zero-emission heavy duty trucks: Battery and Hydrogen Fuel Cell	None	Medium: 31% sales by 2040 and 34% by 2050	High: 67% Sales by 2040 and 69% by 2050
Advanced Low-NO_x CNG Trucks	Displace some diesel trucks	Displace most non-electrified diesel trucks	Displace most non-electrified diesel trucks
Industrial Electrification (non-petroleum)	None	18% of energy consumption by 2050	18% of energy consumption by 2050
Industrial Efficiency (non-petroleum)	BAU	15% reduction in demand from 2015 level by 2050	15% reduction in demand from 2015 level by 2050
Industrial Demand (petroleum)	BAU	Production and demand for in-state extraction and refining reduced by 90% by 2050	Production and demand for in-state extraction and refining reduced by 90% by 2050

Ref: Aas, D. *et al. The Challenge of Retail Gas in California's Low-Carbon Future*. https://ww2.energy.ca.gov/2019publications/CEC-500-2019-055/CEC-500-2019-055-F.pdf (2020).
