# Supplementary material for: Decarbonization will lead to more equitable air quality in California
Source: Nat Commun. 2022 Sep 30;13:5738. doi: 10.1038/s41467-022-33295-9 (PMC9525584; doi:10.1038/s41467-022-33295-9)
Supplement: Supplementary file 2 — Reporting Summary [file 41467_2022_33295_MOESM2_ESM.pdf]

nature portfolio

Corresponding author(s): Scott Samuelsen

Last updated by author(s): Aug 24, 2022

Reporting Summary

Nature Portfolio wishes to improve the reproducibility of the work that we publish. This form provides structure for consistency and transparency in reporting. For further information on Nature Portfolio policies, see our Editorial Policies and the Editorial Policy Checklist.

Statistics

For all statistical analyses, confirm that the following items are present in the figure legend, table legend, main text, or Methods section.

n/a	Confirmed	
☐	☒	The exact sample size (n) for each experimental group/condition, given as a discrete number and unit of measurement
☒	☐	A statement on whether measurements were taken from distinct samples or whether the same sample was measured repeatedly
☒	☐	The statistical test(s) used AND whether they are one- or two-sided<br>*Only common tests should be described solely by name; describe more complex techniques in the Methods section.*
☒	☐	A description of all covariates tested
☐	☒	A description of any assumptions or corrections, such as tests of normality and adjustment for multiple comparisons
☒	☐	A full description of the statistical parameters including central tendency (e.g. means) or other basic estimates (e.g. regression coefficient) AND variation (e.g. standard deviation) or associated estimates of uncertainty (e.g. confidence intervals)
☒	☐	For null hypothesis testing, the test statistic (e.g. F, t, r) with confidence intervals, effect sizes, degrees of freedom and P value noted<br>*Give P values as exact values whenever suitable.*
☒	☐	For Bayesian analysis, information on the choice of priors and Markov chain Monte Carlo settings
☒	☐	For hierarchical and complex designs, identification of the appropriate level for tests and full reporting of outcomes
☒	☐	Estimates of effect sizes (e.g. Cohen's d, Pearson's r), indicating how they were calculated

Our web collection on statistics for biologists contains articles on many of the points above.

Software and code

Policy information about availability of computer code

Data collection	CalEnviroScreen 4.0: a publican mapping tool that identify California disadvantaged communities, published by California OEHHA.<br>E3 PATHWAYS model: a software helps clients plan for and achieve economy-wide decarbonization, published by the Energy+Environmental Economics Company.<br>Mozart V4.0: an offline global chemical transport model particularly suited for studies of the troposphere, published by University Corporation for Atmospheric Research.<br>SMOKEv4.7: an open-source emissions processing system designed to prepare air pollutant emissions data for air quality models inputs. published by the Institute for the Environment U.S.<br>WRF-ARW, 3.7: is an open-source mesoscale numerical weather prediction system published by National Center for Atmospheric Research
Data analysis	CMAQ, v5.2: an open-source multiscale air quality modeling system used for investigation of regional-scale air quality in California published by U.S. EPA.<br>BenMAP-CE, v 1.5: an open-source software that calculates the number and economic value of air pollution-related deaths and illnesses published by U.S. EPA.<br>Matlab v9.8 R2020a: a proprietary multi-paradigm programming language and numeric computing environment developed by MathWorks.<br>ArcGIS 10.5.1: a geographic information system for generating maps ans conducting spatial analysis from Esri.<br>MS Excel Office 365 Version 2207.

For manuscripts utilizing custom algorithms or software that are central to the research but not yet described in published literature, software must be made available to editors and reviewers. We strongly encourage code deposition in a community repository (e.g. GitHub). See the Nature Portfolio guidelines for submitting code & software for further information.

Data

Policy information about availability of data

All manuscripts must include a data availability statement. This statement should provide the following information, where applicable:

- Accession codes, unique identifiers, or web links for publicly available datasets
- A description of any restrictions on data availability
- For clinical datasets or third party data, please ensure that the statement adheres to our policy

All data that support the findings of this study are present in the paper and the supplementary materials, and additional data are available in a publicly accessible repository. The Supplementary Information contains schematic diagram of mitigation pathways, table of scenarios summary of key metrics, emission reductions, conceptual diagram of suits index, and detailed EJ & health benefits allocation comparison for each scenario.The air quality data at 4kmx4km resolution and the health benefits data at the census tract level can be accessed through the public link: https://doi.org/10.17605/OSF.IO/S5RQK.

Field-specific reporting

Please select the one below that is the best fit for your research. If you are not sure, read the appropriate sections before making your selection.

☐ Life sciences ☐ Behavioural & social sciences ☒ Ecological, evolutionary & environmental sciences

For a reference copy of the document with all sections, see nature.com/documents/nr-reporting-summary-flat.pdf

Ecological, evolutionary & environmental sciences study design

All studies must disclose on these points even when the disclosure is negative.

Study description	Our study quantifies the magnitude and distribution of human health benefits under two salient decarbonization pathways (80% below 1990 levels) for California in 2050. This is a 100% numeric modeling study which does not include any form of laboratory or field sample collections. All empirical formula or data used in the model is based on referenced studies.
Research sample	No research sample are included in this study.
Sampling strategy	The method is not used in this study.
Data collection	No data collection performed.
Timing and spatial scale	For numerical modeling. The modeling base year is 2012 (entire year), and the modeling target year is 2050 (entire year), covering the state of California and its neighboring area, at 4km*4km grid.
Data exclusions	No data were excluded from analysis.
Reproducibility	Reproducibility is high with extract raw data and modeling platform. Similar numerical simulations were performed several times per year in our group.
Randomization	No randomization method is used in this study. Covariates control is not relevant to our study.
Blinding	No blinding is used or necessary in this study. For classic numerical modeling, everything is formula based and coded, where blinding is impossible as no human or environmental factor will likely impact the simulation after the calculation starts.

Did the study involve field work? ☐ Yes ☒ No

Reporting for specific materials, systems and methods

We require information from authors about some types of materials, experimental systems and methods used in many studies. Here, indicate whether each material, system or method listed is relevant to your study. If you are not sure if a list item applies to your research, read the appropriate section before selecting a response.

Materials & experimental systems

n/a	Involved in the study
☒	☐ Antibodies
☒	☐ Eukaryotic cell lines
☒	☐ Palaeontology and archaeology
☒	☐ Animals and other organisms
☒	☐ Human research participants
☒	☐ Clinical data
☒	☐ Dual use research of concern

Methods

n/a	Involved in the study
☒	☐ ChIP-seq
☒	☐ Flow cytometry
☒	☐ MRI-based neuroimaging
